# Postpartum vaginal cystic lesions: everyday practice or a differential diagnosis challenge?

**DOI:** 10.1186/2047-783X-18-20

**Published:** 2013-06-26

**Authors:** Nikolaos Machairiotis, Ioannis Tourountous, Alexandros Karamperis, Paul Zarogoulidis, Anastasia Oikonomou, Rokkos Theodoros, Panagiota Palouki, Wolfgang Hohenforst-Schmidt, Athanasios Zissimopoulos, Christodoulos Machairiotis

**Affiliations:** 1 Obstetric Gynecology Department, `Thriassio`` General Hospital, Athens G. Genimata 1, Greece; 2 Pulmonary Department, `G. Papanikolaou`` General Hospital, Aristotle University of Thessaloniki, Thessaloniki, Exohi 1100, Greece; 3 Department of Interventional Pneumology, Ruhrlandklinik, West German Lung Center, University Hospital, University Duisburg-Essen, Essen, Tüschener Weg 40 45239, Germany; 4 Radiology Department, University General Hospital of Alexandroupolis, Democritus University of Thrace, Alexandroupolis, Dragana, Greece; 5 II Medical Clinic, ``Coburg`` Hospital, University of Wuerzburg, Coburg, Ketschendorfer Straße 33, Germany; 6 Nuclear Medicine Department, University General Hospital of Alexandroupolis, Democritus University of Thrace, Alexandroupolis, Dragana, Greece

**Keywords:** Cystic lesion, Labor, Prolapse

## Abstract

Postpartum vaginal cystic lesions constitute a common situation that is caused either by inflammation or by accumulation of lymph. We report a case of a 33-year-old woman who had bilateral duplication of the pelvicalyceal system and ureter, and after the labor of her second child, she had one ureter prolapse into the vagina after initially appearing as a cystic lesion. Ureteral duplication is the most common renal abnormality, occurring in approximately 1% of the population and in 10% of children who are diagnosed with urinary tract infections. In our case we consider possible that this clinical situation was a result of a combination of postpartum pelvic floor trauma and prolapse of the ureter. There are only several of these cases in the literature where ureter prolapse is associated and complicated by pelvic floor trauma caused during or after labor. The clinical approach of the cystic lesions located in the vagina during the postpartum period should include a meticulous examination of the urinary system before any other medical practice.

## Background

Cystic lesions in the vagina after a natural birth are common incidences. The algorithm of the differential diagnosis comprises the inflammation and the formation of a lymphocyst [1]. We report a case of a 33-year-old woman who had bilateral duplication of the pelvicalyceal system and ureter, and she had one ureter prolapse, which appeared as a cystic lesion, into the vagina after the labor of her second child.

## Case presentation

A 33-year-old Caucasian woman was admitted to the outpatient department of our hospital reporting mild pain and discomfort in her vagina after sex. The patient’s obstetrics history included two natural child births - one six months before and the other one two years before. The patient’s physical examination revealed the presence of a bulky, cystic structure located in the left anterior vaginal wall. The patient`s laboratory findings upon admission were white blood count (WBC): 6,900/μl, hematocrit (HCT): 40%, hemoglobin (HGB): 13.2 mg/dL, platelets (PLT): 227,000/μL, glucose: 89 mg/dL, urea: 37 mg/dL, creatinine: 0.7 mg/dL, serum glutamic oxaloacetic transaminase (SGOT): 16 IU/L, serum glutamic pyruvate transaminase (SGPT): 15 IU/L, gamma-glutamyl transpeptidase (γ-GT): 11 IU/L, alkaline phosphatase (ALP): 44 IU/L, Na: 144 mmol/L, K: 4.2 mmol/L, and international normalized ratio (INR): 1.03. The first therapeutic attempt was conservative, using antibiotics, with no improvement of the symptoms. Taking under consideration the fact that the patient did not improve after the medical therapy, paracentesis of the cyst was conducted and the sample was sent for cytological examination, which was negative for the presence of cells. The cystic structure seemed to shrink after each paracentisis followed by an improvement in the patient’s symptoms, but it relapsed after twenty days and the patient experienced the same symptoms accompanied by the presence of fluids in her vagina.

**Figure 1:**
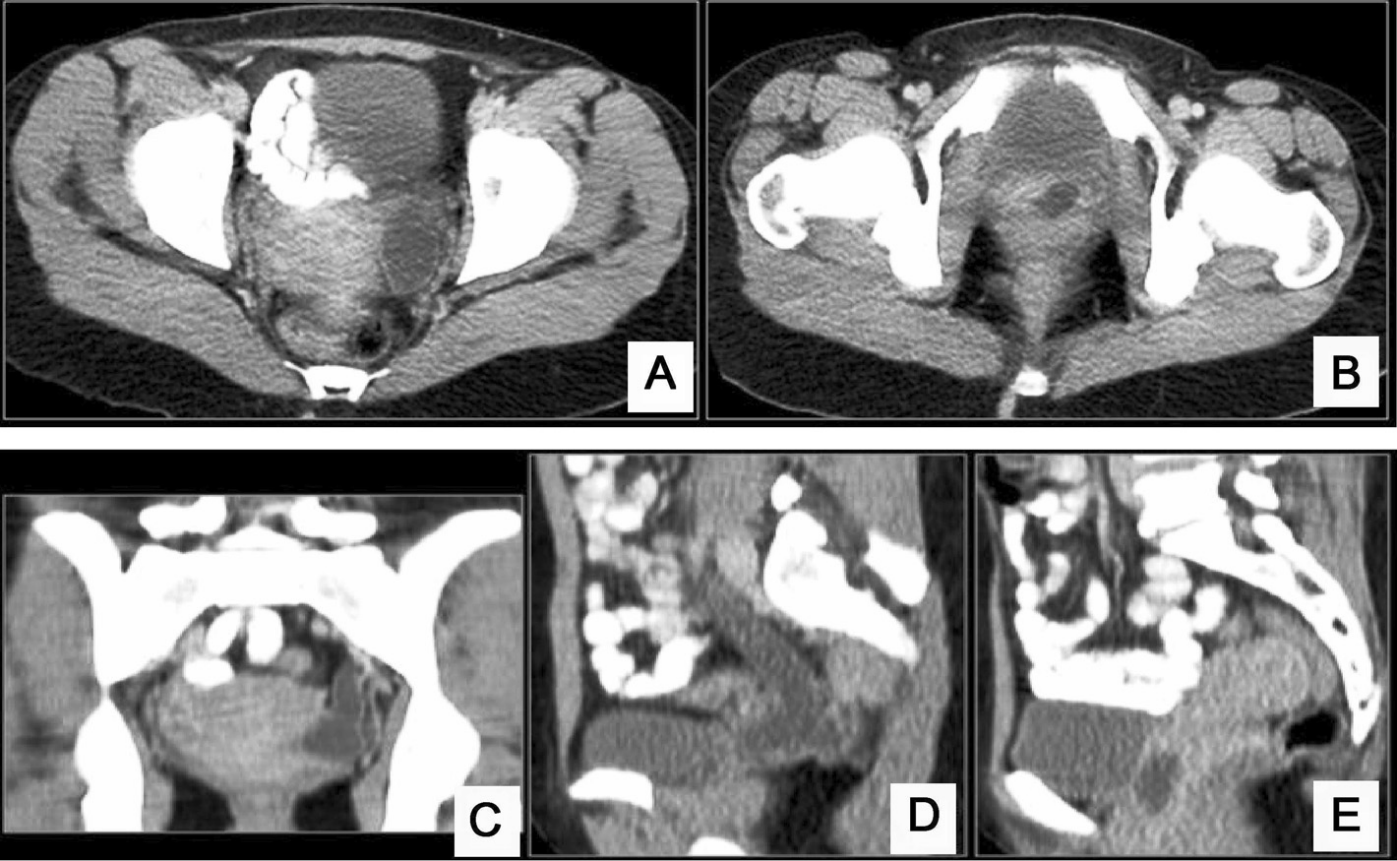
**Computed tomography findings.** Axial contrast-enhanced computed tomography (CT) images of the pelvis show a well-defined oval-shaped cystic lesion at the left of the uterus body (**A**) that extends to the anterior wall of the vagina (**B**). Coronal (**C**) and sequential sagittal (**D**, **E**) contrast-enhanced CT images of the pelvis reveal the tubular morphology of the well-defined cystic lesion at the left of the uterus body (**C**) that extends caudal to the anterior wall of the vagina (**D**) and has a configuration that resembles a ureter.

**Figure 2:**
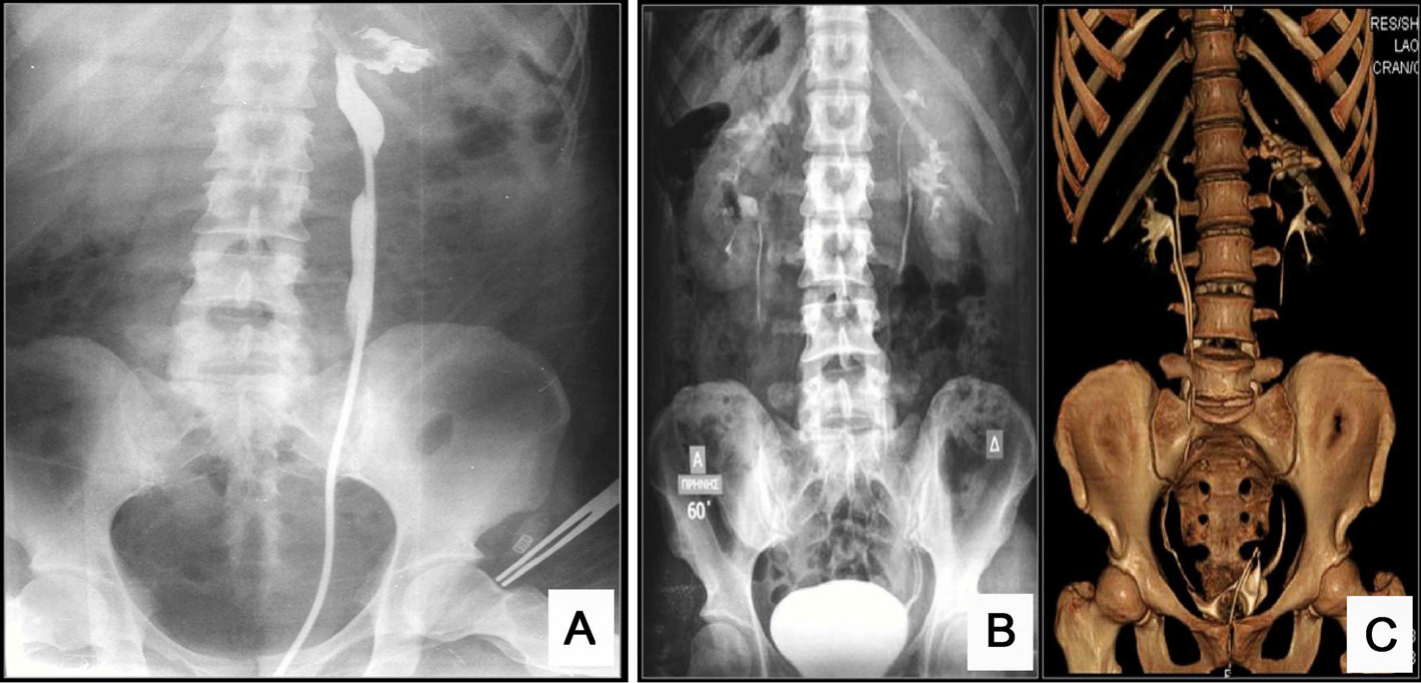
**Intraoperative retrograde pyeloureterography via insertion of Nelaton catheter through the small opening of the intravaginal cystic ‘mass’.** The upper half of a dilated ureter is opacified as well as a dilated pelvicalyceal system at the upper pole of the left kidney (**A**). Excretion urography at 60 minutes post-injection (**B**) and volume rendered computed tomography (CT) urography (**C**) bilateral duplication of pelvicalyceal systems and ureters. The upper pole of the left kidney drained by the ectopic ureter has a grossly dilated pelvicalyceal system (**C**). The ectopic left ureter is no longer dilated.

**Figure 3:**
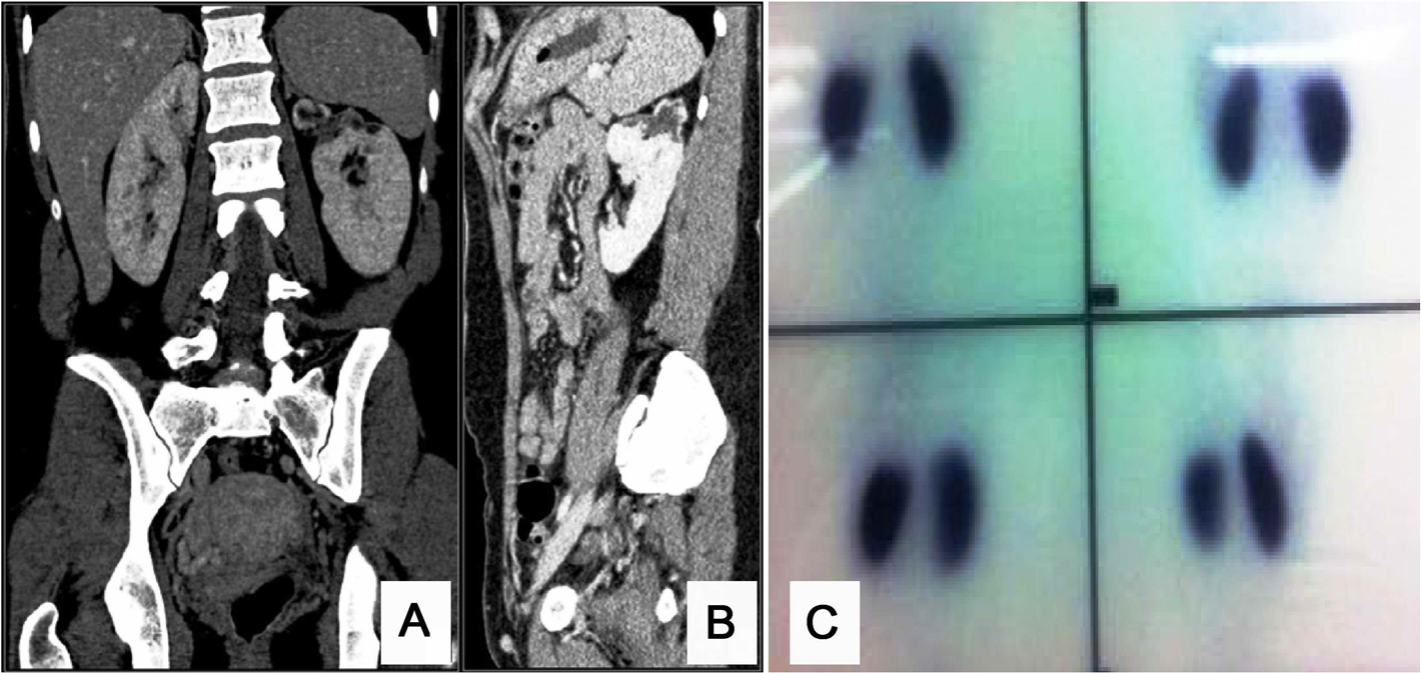
**Computed tomography and V/Q scan after treatment.** Coronal (**A**) and sagittal contrast-enhanced computed tomography (CT) images (**B**) reveal the cystic degeneration of the upper pole of the kidney drained by the chronically obstructed ectopic left ureter, ^99m^Technetium- dimercapto-succinic-acid (^99m^Tc-DMSA) renal scintigraphy (**C**) reveals foto-deficient area, located on the upper pole of the left kidney.

The patient’s work-up included a computed tomography of the lower abdomen which revealed a well-defined, oval shaped cystic lesion on the left of the uterus body (Figures 1 and 2). After that, a surgical excision of the lesion was scheduled. During the operation rupture of the cystic structure occurred, followed by its partial excision and injection of methylene blue in the bladder in order to clarify if this lesion communicated with it. The methylene blue test was negative, but there was outflow of serous fluid from the lesion, and there was also a small orifice on its top. A small Nelaton catheter was inserted through the orifice and contrast agent was injected followed by an X-ray, which showed that the anatomy of lesion was compatible with that of a ureter. The surgical specimen was sent for histological examination, which confirmed its ureteral origin. Additionally, a pouch between the cystic structure and the vagina was created, which led to the outflow of urine from the vagina. During the surgery the patient underwent to a pyeloureterography via insertion of Nelaton catheter through the small opening of the intravaginal cystic ‘mass’ and according to these findings, underwent a computed tomography (CT) urography and a CT of the upper and lower abdomen on the same day (Figure 2), which were repeated 20 days after that (Figure 3). Imaging tests revealed that the patient had a bilaterally duplicated ureter. A ^99m^Technetium-dimercapto-succinic-acid (^99m^Tc-DMSA) was performed in order to clarify the renal function of the patient. Renal scintigraphy (anterior, posterior, right anterior oblique (RAO) and left anterior oblique (LAO) images) with a gamma camera (Millenium MPR; General Electric, Milwaukee, Wisconsin) was performed 4 hours after the intravenous injection of 4 mCi (148 MBq) technetium-99 dimercaptosuccinic acid (DMSA). The patient’s renal scintigraphy (^99m^Tc-DMSA) showed a hydronephrotic lesion, which presented as a wide foto-deficient area, located on the upper pole of the left kidney (Figure 3). The woman underwent nephrostomy, and two months later the vagina wall was sutured. The patient`s laboratory findings upon discharge were as follows: WBC: 8,600/μl, HCT: 34.8%, HGB: 11.4 mg/dL, PLT: 219000/μL, glucose: 81 mg/dl, urea: 33 mg/dL, creatinine: 0.6, Na: 139 mmol/L, K: 4.2 mmol/L, INR: 1.05, SGOT: 18 IU/L, SGPT: 14 IU/L, γ-GT: 11 IU/L, and ALP: 44 IU/L.

## Conclusions

The congenital abnormalities of the ureters constitute a complex and wide field of urology. In the past the detection of these anomalies was hampered by the lack of appropriate imaging techniques available for the manifestation of these abnormalities. The duplication of the ureter is the most common renal abnormality. It affects approximately 1% of the population and 10% of children who suffer from urinary tract infections [2]. The ureter duplication can be either complete or incomplete. Complete ureteral duplication is the situation in which two ureters enter the bladder and increase the possibilities of vesicoureteral reflux into the lower pole and obstruction to the upper pole of the kidney [3]. On the other hand, incomplete ureteral duplication is a clinical situation in which one common ureter enters the bladder and this situation is rarely clinically significant [3]. Additionally, the upper pole ureter may be ectopic in its insertion into the bladder or it may end in the formation of a ureterocele. These two conditions are more common in duplicated collecting systems than in single systems.

In order to understand this situation it is crucial to refer to the ureteral embryology. Ureteral development begins at the fourth week of gestation. More specifically, the ureteral bud branches off of the mesonephric (or Wolffian) duct and eventually extends into the nephrogenic blastema. Embryologically, the ureteral bud is responsible for the formation of the renal collecting system, beginning at the ureteral orifice and ending at the collecting ducts of the kidney. The mesonephric duct is incorporated into the bladder at the locum of the distal aspect of the ureteral bud, the mesonephric duct, which is accompanied by the location of the ureteral orifice superolaterally, taking its normal position on the trigone, whereas the more distal segment of the mesonephric duct is carried inferomedially and is incorporated into the bladder neck. In females, the distal segment of the mesonephric duct forms the Gartner duct, which is located between the vagina and urethra [4].

In cases of ureteral duplication, where the ureteral bud is formed twice, the ureter of the lower part integrates with the bladder earlier than expected and, as a result, is carried into a more superolateral position; this results in the distal ureter being poorly supported by the trigone and having a shorter intramural tunnel. These situations increase the likelihood of vesicoureteral reflux [5]. Thus, the upper pole ureter integrates with the bladder later than usual and is inferomedially carried, thereby increasing the possibility of the presence of a ureteral orifice that is either located in a lower position on the trigone or is ectopically found at the bladder neck or ejaculatory duct. In females, the ureter may end in a Gartner duct, resulting occasionally in the erosion of the nearby vagina or the urethra, inferiorly to the urinary sphincter. This is considered to be the cause of continuous urinary incontinence in females [5].

In our case we consider the possibility that this clinical situation was a result of a combination of postpartum pelvic floor trauma and prolapse of the ureter. There are several cases in the literature referring to pelvic floor trauma caused during or after the labor [6]. More specifically, there have been studies in which parity has been identified as a risk factor for pelvic organ prolapsed [6-10]. Females with one vaginal child birth had 4 times and women with two child births an 8.4 times greater possibility of subsequently requiring admission to the hospital for prolapsed pelvic organs [11,12]. Avulsion of the puborectalis and the levator muscle is considered to be responsible for a prolapsed pelvic organ after delivery [13,14].

However, apart from avulsion or macrotrauma, other forms of pelvic floor injury may play a role in the pathogenesis of pelvic organ prolapse. The levator hiatus, is the anatomical area that is found between the arms of the V. It contains the urethra anteriorly, the vagina in the center and the anorectum posteriorly. This area varies from 6 to 36 cm^2^ according to the Valsalva maneuver in young nulliparous women [15]. Taking under consideration the fact that the space needed for the average fetal head in the plane of minimal diameters is 70 to 100 cm^2^ (after the equation to a head circumference of approximately 300 to 350 mm), the distension and deformation of the levator complex is inevitable [15]. A study of Shek *et al*. on primiparous women pre- and postpartum has proven an increase in hiatal area during Valsalva maneuver by 6% without any evidence of gross levator trauma. These results suggest that traumatic overdistension or ‘microtrauma’ could be an alternative type of levator injury. Although there is limited research on levator overdistension, significant correlations have been found as far as the area of the levator hiatus and pelvic organ descent are concerned, both in symptomatic and asymptomatic women [16]. All these changes that occur in the pelvis floor and the vagina after labor can explain the presence of the ureter in the vagina. In fact we consider possible that the prolapse of the left upper pole ureter into the vagina in the case presented is caused by the trauma of the muscles that either support or form the vagina.

The management of the ectopic ureter consists of surgical reconstruction. The approach depends on whether the system is duplex or single, and the extent of function of the involved kidney moiety. Because most cases of ectopic ureter in a duplex system are associated with a dysplastic upper pole renal segment, the excision of this segment along with the proximal ureter is usually curative. In cases where the upper pole has good function, the ectopic ureter is re-implanted into the bladder or anastomosed outside the bladder to the normal pole ureter (ureteroureterostomy) [17]. The therapeutic approach in patients with a single system in which the kidney is usually dysfunctional and the base of the bladder (hemitrigone) is poorly developed treatment usually consists of nephrourecterectomy. If the kidney is functional, the treatment is resection of the distal ectopic ureter and re-implantation into the bladder [17,18].

In conclusion, it is crucial for the obstetricians-gynecologists to be aware of the various congenital abnormalities of the urinary tract that may alter the anatomy of the pelvis. The clinical approach of the cystic lesions located in the vagina during the postpartum period should comprise an assiduous examination of the urinary system before any other medical practice. As a consequence of the clinical approach mentioned above, we suggest the inclusion of the specific imaging analysis of the anatomy and function of the urinary tract (CT-urography, intravenous pyelography) in the algorithm that is used for the differential diagnosis and management of postpartum vaginal cystic lesions.

## Consent

Written informed consent was obtained from the patient for publication of this Case report and any accompanying images. A copy of the written consent is available for review by the Editor-in-Chief of this journal.

## Abbreviations

ALP: Alkaline phosphatase; CT: Computed tomography; DMSA: Dimercaptosuccinic acid scan; HCT: Hematocrit; Hgb: Hemoglobin; INR: International normalized ratio; LAO: Left anterior oblique; PLT: Platelets; RAO: Right anterior oblique; SGOT: Serum glutamic oxaloacetic transaminase; SGPT: Serum glutamic pyruvate transaminase; WBC: White blood count; γ-GT: Gamma-glutamyl transpeptidase.

## Competing interests

The authors report no conflicts of interest. The authors alone are responsible for the content and writing of the paper.

## Authors’ contributions

NM and IT made the same contributions to this paper. These authors collected the data of the patient and contributed in the writing of the manuscript. AK, RT and PP diagnosed and treated the patient. AO evaluated the radiologic findings. AZ performed and evaluated the DMSA exam and results. PZ, NM and WH-S performed the editing and coordinated the collaboration between different centers for external evaluation. CM is the director of the department, treated the patient and provided useful insights. All authors read and approved the final manuscript.
